# Scaling the mountains: what lies above 7 Tesla magnetic resonance?

**DOI:** 10.1007/s10334-023-01087-x

**Published:** 2023-04-19

**Authors:** Rita Schmidt, Elena Kleban, Saskia Bollmann, Christopher J. Wiggins, Thoralf Niendorf

**Affiliations:** 1 Department of Brain Sciences, Weizmann Institute of Science, Rehovot, Israel; 2 Department of Diagnostic, Interventional and Pediatric Radiology, Inselspital, University of Bern, Bern, Switzerland; 3 School of Information Technology and Electrical Engineering, Faculty of Engineering, Architecture and Information Technology, The University of Queensland, Brisbane, Australia; 4 Imaging Core Facility, Institute for Neurology and Medicine, Forschungszentrum Julich, Julich, Germany; 5 Berlin Ultrahigh Field Facility, Max-Delbrueck Center for Molecular Medicine in the Helmholtz Association, Berlin, Germany

## Foothills: where are we coming from

The progress of ultrahigh-field magnetic resonance (UHF-MR) provides meaningful technologies for the advancement of biomedical and diagnostic MRI. The argument for moving 7 T MRI into clinical applications is more compelling than ever. Images from these instruments have revealed new aspects of anatomy, function and physio-metabolic characteristics of the neuro, neurovascular, cardiovascular, musculoskeletal, renal, hepatic and ocular systems, as well as other organs and tissues with unparalleled detail. UHF-MR has shown an amazing spectrum of potential clinical uses, with implications for neuroscience, neurology, radiology, neuroradiology, cardiology, internal medicine, oncology, nephrology, ophthalmology and many other clinical fields [1–7].

## Getting ready to leave base camp

With 7 T human MRI now present in the clinic, there is increasing interest in exploring ever higher magnetic field strengths. This includes pioneering reports on 9.4 T, 10.5 and 11.7 T MRI which lead the way to even higher fields [8–10]. This has generated the momentum to drive MR science to take even more ambitious steps into the future, envisioning human MR at 14 T and even 20 T [11–15]. The world’s first whole-body MR scanner was the Aberdeen Mark 1, with *B*_0_ = 0.04 T [16]. Ascending from this point to the alpine heights of 14 T or 20 T within about 40–45 years entails a 350–500-fold increase—an extraordinary climb, and a testament to the endurance and passion of the pioneers and highly creative interdisciplinary teams of explorers. Nevertheless, considerable challenges still thwart the push beyond current magnetic field boundaries. These challenges are both practical (expense!), as well as technical, especially when going above 12 T due to the requirements for alternative superconductor technologies. Each step upward will require rigorous technical and in vivo studies, and the route has to be tested by those who adapt the technology. Fortunately, recent experience at 7 T encourages us that such efforts will be worthwhile. How will we get to these dizzying heights of B_0_ > 7 T and what will be the enabling technologies? What are the expected scientific benefits that will come from this? How will climbing to higher field strengths broaden the horizons of MR applications? Moving to 7 T has shown concrete benefits compared to 3 T for several clinical indications, including epilepsy lesion detection, diagnosis of neurodegenerative and neuroinflammatory disorders and assessment of musculoskeletal diseases [17–19]. Will these indications yield further benefits at higher fields? For other indications, the current belief is that detection levels and clinical benefits remain just sub-threshold with 7 T MRI; will these become clinically relevant at higher fields? How can neuroscience continue to make giant strides towards deciphering the human brain by bridging the gap between histology, neurochemistry, neurobiology and in vivo MRI? Can we learn to make more sense of the terrain we currently inhabit at 7 T and lower fields, and better interpret the biological, physiological and metabolic meaning of the imaging data we obtain at higher fields? Can we close the spatial resolution gap which is still wide open at 7 T and below, compared to optical imaging? How can we address safety implications of higher fields, for both the subject and the staff? How can we leverage the recent progress and novel approaches of materials science to overcome technical barriers and reduce the expense of higher field systems? How can we resolve the cost–benefit argument, to ensure that ultrahigh-field MRI is a technology that yields real results for the community, and does not turn out to be a sophisticated research toy? Technical gaps, resolution gaps, public communication gaps—these are crevasses that we must traverse along our climb up the mountain.

## Mountaineering hardware and equipment

*Life's a bit like mountaineering—never look down.* – Edmund Hillary

Realising the breadth of challenges and the rich opportunities for discovery, this Special Issue is very timely. Our goal is to guide the field by attempting to answer some of these questions and to explore what lies in this new mountainous terrain of MRI above 7 T. Climbing up the UHF mountain, it became clear that the field strengths of 7 T, 8 T and 9.4 T were just ridges, and that further climbing was in store. However, at 10.5 T and 11.7 T, questions arise: What lies above? Is there another ridge or summit, where we can explore projects at 14 T? And will this be a plateau, with nothing more possible above, or rather just another in a series of ridges? And, once we have reached this level, will there be an influx of explorers testing the limits and translating the benefits to society at large, or rather will the air at this altitude be so thin that it can sustain only a select few who have access to the technology suitable to explore these dizzying heights? What we know for sure is that challenges and requirements of 14 T MR will pave the way for further advances in MR technology, MR systems design and MR magnet design. Freeman Dyson, a mathematical genius turned into a technological visionary who described the dynamics of technology development and scientific discovery in his book “*Imagined Worlds*” offers us some inspiration: “*New directions in science are launched by new tools much more often than by new concepts, the effect of a concept-driven revolution is to explain old things in new ways. The effect of a tool-driven revolution is to discover new things that have to be explained”*. [20].

## About magnets and mountains

An initial reaction upon looking at the massif of the Iseult 11.7 T system and the 14 T proposals might well be that such high field systems are too complicated, too difficult to fabricate and too physically large to be installed in multiple facilities around the world, let alone in clinical settings. Similar concerns about 7 T systems were raised in the past. Examining the history of 7 T magnet technology shows that the first generation of passively shielded magnets (length = 336 cm) was replaced by the first generation of actively shielded 7 T magnets (length = 255 cm), which were then superseded by the next generation of actively shielded magnets (length = 270 cm, weight less than 25 t, minimum room size 65 m^2^), demonstrating how developments in magnet technology can easily move towards smaller, lighter and more clinically oriented systems. Pioneers from the MR research and superconducting materials science fields have already taken bold steps into higher altitudes, designing superconducting magnets customised for 14 T whole-body MRI [21, 22]. One high-temperature superconductor (HTS)-based design has a very compact configuration (total weight: 15 t, length: 1.9 m, outer diameter, 1.3 m, total HTS wire consumption of 455 km, weight of the HTS wires: 8.4 t, warm bore 80 cm) [21]. This compact magnet design would be even smaller and lighter than current commercial 7 T instruments. An open minded look reveals that the development of HTSs and magnet design for 14 T class human MR instruments is in a creative state of flux. Still, what has been learned about magnet designs at 14 T, will inevitably be applied both to 20 T class instruments, but also to 7 T and lower magnetic field strengths, once HTSs become more affordable.

## Sending and receiving signals during the climb

To harness the potential of higher field MRI systems, substantial efforts have been dedicated to developing radiofrequency (RF) coil technology and related methodology that can cope with the challenges of RF transmission field inhomogeneity and increased RF power deposition. A large body of work has already provided enormous progress and results that robustly demonstrate optimal and safe human MR scanning at 7 T, 9.4 T and 10.5 T [8, 23–27]. This includes implementation of multi-channel transmit-receive RF arrays, approaching the ultimate signal-to-noise ratio, improvements in RF transmission field distribution and enhanced SAR prediction and management [28]. The tools to implement and use parallel transmission methods, like the kT points approach, are now well documented, enabling easier implementation and dissemination across a wide range of sites and users [29–31]. Novel RF array designs include optimal combinations of loop-dipole building blocks, as well as dielectric resonators, with numerous studies demonstrating their added value at 7 T [32] and 10.5 T. This Special Issue highlights the merits of transmit RF arrays. Configurations using 2, 8, 16, or 32 transmit channels were successfully utilised for a spectrum of applications, ranging from functional brain mapping at 9.4 T with 1 mm spatial resolution [33] to high spatial and temporal resolution spin-echo line scanning that ensures microvascular specificity of functional responses [34], as well as high spatiotemporal resolution quantification of near-wall haemodynamic parameters in in vitro intracranial aneurysms [35], resolving wall shear stress patterns, and cardiac and body imaging in humans and large experimental models at 7 T [36] and 10.5 T [8].

As we move up the mountain face, we should pause for a moment to acknowledge and applaud the pioneers who climbed the very steep mountains to 9.4 T [33], 10.5 T [8, 37, 38] and 11.7 T [39]. These explorers were essentially free climbing, reaching the summit without vendor-provided RF coils and without push-button software, yet nevertheless navigating through the enormous challenges towards beautiful images of the brain and the body enabled by parallel transmission. First MR images obtained at 11.7 T of an ex vivo brain are reported in this Special Issue, and the imaging community is excited to hear about the next achievements [39].

Without pausing further, we should take a deep breath, and approach the next mountain before us. A study in this Special Issue provides new insights on simulation-based evaluation of RF coils applicable for brain imaging at 14 T [40]. Although there are still open questions about increased SAR, the study shows prospective practical and favourable implementation based on 16 transmission channels. Electromagnetic field simulations using high-density head RF arrays demonstrated that transmission fields suitable for ^1^H MR of the human brain can be achieved at 1 GHz (23.5 T) [14]. The calculated relative increase in SAR at 23.5 T versus 7.0 T was reported to be below 1.4 (in-phase phase setting) and 2.7 (circular polarised phase setting) [14]. Imaging of the human body and heart at 14 T is also conceptually appealing because it provides rich opportunities for discovery into cardiovascular health and disease. Nurzed et. al. show in this Special Issue that MRI of the human heart at 14 T is feasible, by integrating high-density RF antenna arrays and parallel transmission, thus increasing the degrees of freedom for tailoring the excitation profile and minimising RF power deposition [41]. These insights provide a solid technical foundation for further explorations into cardiac and body MR at 14 T and will ultimately help to unveil new dimensions of the processes of health and disease.

## Rich opportunities for discovery

*I think it all comes down to motivation. If you really want to do something, you will work hard for it.* – Edmund Hillary

The review reports in this Special Issue “A vision of 14 T MR for fundamental and clinical science” [42] and “Germany’s Journey Toward 14 Tesla Human Magnetic Resonance” [43] discuss the potential of RF coil design tailored for 14 T. Amongst the key challenges ahead are multi-nuclei RF coils, which open new frontiers for physio-metabolic probing with X-nuclei MR (e.g. deuterium and carbon) using shorter scan times—even clinically acceptable scan times. In fact, the resonance frequencies of physiologically relevant X-nuclei at 14 T and 20 T are below the ^1^H resonance frequency at 7 T, with the exception of ^19^F, ^3^He and ^31^P (20 T). This makes RF technology established for ^1^H MR at 7 T very well suited to be adapted and fine-tuned for hetero-nuclear MR at 14 T or 20 T. Leveraging this advantage, MR at 14 T or 20 T is conceptually appealing to improve our understanding of ion homeostasis and energy metabolism in vivo in humans, as outlined in [43]. For example, the sensitivity gain at 20 T is expected to reduce scan times for ^31^P and ^23^Na by a factor of 8–10 versus approaches outlined in this Special Issue for 7 T [44]. Implications of these gains in sensitivity and speed include the promise of sodium MR of the heart with a sub-millimetre spatial resolution in 5–10 min scan time, and the potential for probing cardiac metabolism with ^31^P MR spectroscopy in clinically acceptable examination times. This is in stark contrast to scan durations of approximately 1–2 h available today for the same sub-millimetre spatial resolution [45–47]. MR of nuclei such as ^35^Cl and ^39^ K will greatly benefit from 14 and 20 T. Arguably, ^39^ K MR remains quite challenging because the sensitivity is about six orders of magnitude less than that of ^1^H MR [48]. Notwithstanding this constraint, it stands to reason that ^39^ K MR at 14 T or 20 T will enable, for the first time, quantitative in vivo assessment of myocardial potassium content on a cellular level, which would open an entirely new research field of MRI-based physio-metabolic fingerprinting as a link to personalised medicine, as pointed out in this Special Issue in [43].

As we continue uphill, there is also a place for new opportunities and disruptive approaches which go beyond traditional RF coil design. One intriguing trajectory includes structures based on metamaterials to better manage RF wave propagation [49]. What can this approach offer to the high field climbers? More efficient RF coils? Flexible and dynamic change of the RF field distribution? Simple multi-band design? Reduction in RF power deposition? Whether with regard to the pace of progress, potential discoveries, or clinical applicability, the only sure thing about predicting the future of high-field MRI technology is that we will almost certainly underestimate the game-changing potential of these synthetic material structures to enable advanced RF coil designs. New opportunities may also lie away from the conventional beaten track. One might imagine, is homogenization of the RF field what we are really after? Could we instead exploit inhomogeneous RF fields and use it as an advantage? A well-known and recently FDA approved example is the MR fingerprinting approach which deploys a set of inhomogeneous RF fields as part of the design for fast scan implementation [50]. Whilst in vivo applications of MR above 7 T already can benefit from the accumulated knowledge and progress in RF coil technology, this Special Issue also highlights that we still have new adventures ahead.

## Avoiding avalanches—safety comes first

As with the advent of any new biomedical imaging technology, pushing the boundaries of magnetic field strengths above 7 T presents safety challenges that must be carefully and rigorously tested for their potential physiological impact and physical effects. These assessments need to be supported by a harmonisation of the technical standards and guidelines [51], to lift the limits on the static magnetic field strength for the first level controlled operating mode from 8.0 T to 14.0 T, taking into account the state-of-the-art in the scientific literature. One important consideration at these dizzying heights above 7 T is exactly that the well-known vestibular effects that are provoked by movement through or static positioning within the field of these magnets. whilst these have generally been adequately addressed by appropriate subject positioning and handling (e.g. speed of the patient bed), it is possible that subject tolerance of these effects may become a limiting factor in applications in humans at higher magnetic fields [52].

## Pioneers, explorers and early adopters

*I think the really good mountaineer is the one with the technical ability of the professional and with the enthusiasm and freshness of approach of the amateur.* – Edmund Hillary

If RF engineers and magnet designers are the Tenzing Norgays and Edmund Hillarys of UHF-MR, neuroscientists are the MR equivalent of the 1963 Everest Expedition, which, in a tremendous team effort not only put six people on the mountain, but also performed scientific experiments in this challenging environment. The appeal for neuroscience at 7 T and above is the substantial increase in signal-to-noise and contrast-to-noise ratio, which is usually traded for higher spatial resolution. Both papers in this Special Issue which speak about 14 T MR instruments aim to resolve anatomically and functionally the layers of the cortex, and to reach the intrinsic vascular limit of fMRI: the capillary spacing on the order of 40–50 µm. This could open up new views of neural communication and feedforward and feedback loops as outlined in [42] of this Special Issue. Similar to the early mountaineers who relied on the help of the Sherpas, neuroscientists need ingenuity, endurance and faithful collaboration with the locals (MR physicists, mathematicians, data scientists, expert engineers and other related experts) to fully realise the potential of MRI above 7 T. This may include changing from 2D EPI to 3D EPI acquisitions to limit SAR, the development of dedicated head magnetic field gradients to achieve the necessary short readout times, or revisiting existing imaging principles to achieve high spatial and temporal resolution. One example is provided by Raimondo et al., who paved the way for spin-echo line scanning, which can achieve spatial and temporal resolutions of 0.25 mm and 200 ms, respectively as pointed out in [34] of this Special Issue. Alternatively, highly anisotropic FLASH acquisitions can provide spatial resolutions of 0.1 mm along one axis, to match the columnar or laminar structure of the cortex as demonstrated in [33] of this Special Issue. When leaving the well-trodden path of ‘functional’ MRI, ultrahigh-field MR scanners also offer an increase in spatial and spectral resolution to investigate brain metabolism and neuro-metabolites such as GABA or glutamine and glutamate [42], and can provide incredibly detailed post-mortem brain images [33]. Given how busy Everest is nowadays, we hope that these first experiments are similarly just the beginning of a thriving and innovative neuroscience community exploring the human brain using the vast potential of ultrahigh-field MRI.

## Pushing the limits

Climbing up mountains above 7 T is more than just a matter of designing new magnets and RF coils, and installing them and trying to operate them in "core facilities", equipped with the concepts that have guided research and discovery at lower field strengths. The ultimate game-changing potential of 14 T technology is far greater. However, realising this potential requires that we address candidly the question of whether 14 T MR stands a realistic chance to become a new diagnostic tool, or rather turn out to be just an expensive research toy for a minority of biomedical engineers and scientists. History again offers a lesson. When the first in vivo human scan was performed at 7 T, it seemed unimaginable that just 20 years later 7 T scanners would be approved for clinical use for the brain and extremities. However, the answer to the question of the utility to the public of 14 T MRI is not a foregone conclusion. Certainly, the higher spatial and spectral resolution, and superior tissue contrast at 7 T versus lower field counterparts make it particularly attractive for clinical diagnostics and scientific applications. These advantages of even higher field strengths have the potential to reveal smaller anatomical features and even better diagnostic accuracy, which could benefit patients greatly—still the clinical utility must be demonstrated. What makes a medical device fit for clinical use? It has to be safe, consistent in its performance and effective in diagnosing medical conditions. With regulatory clearance of 7 T MRI scanners already established, the safety and technical reliability criteria can be considered as fulfilled. However, a full reckoning of the clinical advantages of 7 T MRI over lower magnetic fields remains to be seen. It is crucial to identify applications where the benefits of using 7 T MRI outweigh the increased costs, and where earlier and more precise diagnoses can improve patient outcomes. Only then can we justify the utilisation of 7 T instead of 3 T for selected applications, and then think about going to higher field strengths. This is not just a question for MR scientists and clinicians, but also for the public at large, and their political representatives, who must balance competing demands for limited financial resources for publicly funded healthcare systems as highlighted in [43] of this Special Issue. Before we can bridge that communication crevasse and explain to the politician why it is a good idea for the government to invest in ultrahigh-field MRI systems, we need to have a good answer to that question ourselves. No one said climbing up the mountain would be cheap!

## Approaching the summit

From the foothills, the base camp of today at 7 T MR may already look like a mountainous height. Inevitably, this will be the low field of tomorrow. Ambitions towards 14 T are close to becoming the new reality [42, 43]. And there is amazing news—in early 2023, the *Dutch National 14 Tesla Initiative in Medical Science* (DYNAMIC) has received funding for implementation of the first 14 T class human MR instrument as part of the large-scale research infrastructure national roadmap of the Netherlands [53]. What can we gain by going to such high magnetic fields? The obvious gain in sensitivity will allow us to spatially resolve tissue at even finer biologically meaningful scales, opening up a plethora of possible applications in fundamental research. One research area which will undoubtedly derive tremendous benefit from going to 14 T is physio-metabolic probing with X-nuclei MR. But first and foremost, we need the research to focus on preparing the appropriate hardware for climbing such a high peak. For some applications at 7 T, one can still tolerate the image quality resulting from single transmit channel excitation; at 14 T, parallel transmission will become essential, not only for ^1^H but also for hetero-nuclear MR. You can read more about what research groups envision to achieve at 14 T in the extended commentary [42] and in the review [43] of this Special Issue.

## Standing at the pinnacle and looking beyond the horizon

*While on top of Everest, I looked across the valley towards the great peak Makalu and mentally worked out a route about how it could be climbed. It showed me that even though I was standing on top of the world, it wasn't the end of everything. I was still looking beyond to other interesting challenges.* – Edmund Hillary

The regulatory approval of 7 T scanners has paved the way for their wider use in clinical settings, and ongoing research may uncover further applications where 7 T MRI can be fruitful. What can we expect to happen in the next 20 years? As we see from the current interest in low field MR, nothing ever dies. We just develop a broader range of tools, each best suited to a particular question. Will 7 T replace 3 T, and will 14 T become the new lower boundary for ultrahigh-field MR? Or will we be able to even double the maximal clinical magnetic field strength? Though the approach to that summit will be difficult, the view from above the clouds will be unparalleled, and that view always makes the climb worth the effort. With every milestone of improved resolution, speed, or contrast that we pass by along the ascent, new routes are opened up for basic research including technology development, numerical simulations, state-of-the-art machine learning approaches, tissue specimens and animal models and applications in the clinic.

There is a bustling atmosphere and a restless energy at basecamp. The work presented in this Special Issue provides convincing reasons that there is no turning back on the Tesla road scaling the mountains. But we need sure footing to avoid the threat of loose snow and avalanches: the ongoing challenges should be communicated and addressed openly in collaborations between forward-thinking researchers with the diverse expertise that has gotten the field this far. This calls for new concepts for research frameworks, international partnerships and collaborative culture, to provide a springboard for image makers to peer deeper and deeper into healthy and diseased tissue to connect views across length and time scales. This is in the spirit of the physicist Richard Feynman who highlighted the importance of imaging in his landmark lecture in 1959 by stating *“that it is very easy to answer many of these fundamental biological questions; you just look at the thing!”.* Let’s climb the mountain and take a look.

## Data Availability

No datasets were generated or analyzed during the current study.
