# Correction to: Scaling the mountains: what lies above 7 Tesla magnetic resonance?

**DOI:** 10.1007/s10334-023-01097-9

**Published:** 2023-05-16

**Authors:** Rita Schmidt, Elena Kleban, Saskia Bollmann, Christopher J. Wiggins, Thoralf Niendorf

**Affiliations:** 1 Department of Brain Sciences, Weizmann Institute of Science, Rehovot, Israel; 2 Department of Diagnostic, Interventional and Pediatric Radiology, Inselspital, University of Bern, Bern, Switzerland; 3 School of Information Technology and Electrical Engineering, Faculty of Engineering, Architecture and Information Technology, The University of Queensland, Brisbane, Australia; 4 Imaging Core Facility, Institute for Neurology and Medicine, Forschungszentrum Julich, Julich, Germany; 5 Berlin Ultrahigh Field Facility, Max-Delbrueck Center for Molecular Medicine in the Helmholtz Association, Berlin, Germany

**Correction to: Magnetic Resonance Materials in Physics, Biology and Medicine (2023) 36:151–157** 10.1007/s10334-023-01087-x

The original version of this article unfortunately contained a mistake. The author’s name Elena Kleban was incorrectly written as Elena Keban.

The original article has been corrected.

